# Oral health needs and case mix complexity of adults with disabilities requiring special care dentistry: a retrospective hospital audit

**DOI:** 10.3389/froh.2026.1854266

**Published:** 2026-07-08

**Authors:** Gabriel Keng Yan Lee, Chong Meng Tay, Claire Zhen Yi Cheong, Yu Fan Sim, Christopher Peck, Mathew Albert Wei Ting Lim

**Affiliations:** 1 Faculty of Dentistry, National University of Singapore, Singapore; 2 National University Centre for Oral Health Singapore, Singapore; 3 Melbourne Dental School, University of Melbourne, Carlton, Victoria, Australia

**Keywords:** case mix, disability, hospital records, oral health, special care dentistry

## Abstract

**Introduction:**

Adults with disabilities may present with conditions that exacerbate symptoms of oral diseases, resulting in changes in behaviours and distress. Access to necessary oral healthcare is often available at various levels of the healthcare system, and the appropriate level of care may be stratified according to case complexity. This study was conducted in a tertiary-level centre for oral health that provides specialist-level care for adults with disabilities and special needs, often referred from primary care or other tertiary healthcare services.

**Methods:**

Analysis of patients' anonymised first visit hospital records from March 2022 to December 2024 included medical diagnoses, oral status, indication for the need for advanced pharmacological behavioural management (i.e., sedation, general anaesthesia), and written notes. Case mix complexity was scored through reviewer analysis of each record.

**Results:**

184 records were included in the analysis. The largest source of referrals was from tertiary medical services (43.9%), and the most common disability was that of mixed disability (34.8%). 33.2% of patients presented with active dental caries, and advanced pharmacological behavioural management was indicated in 37% of cases. 48.4% of cases were classified as Severe Complexity, and 10.9% as Extreme Complexity. The study also found indications that higher care needs were from patients referred from paediatric services and adults with intellectual disability.

**Conclusion:**

Within this study population of patients attending tertiary oral health services, oral health needs and case complexity is high. The analysis of hospital records provides valuable insight for resource allocation and strategic initiatives. This provides supporting evidence to inform efforts aimed at improving integration of care pathways for these population segments.

## Introduction

Disability is broadly defined by the World Health Organization as the interaction between individuals with a health condition and personal and environmental factors (1). Adults with disabilities may present with conditions affecting their intellect, physical function, social interaction, and mental wellbeing. Persons with such lived experiences often face significant barriers in accessing oral healthcare services, such as logistical issues, financial constraints, and the lack of appropriate services (2). As a result, the oral health of this demographic tends to have higher rates of periodontal disease and dental caries (3), thereby having a larger impact on overall health and well-being compared to persons without disabilities. Such oral diseases are also known to lead to symptoms such as pain, which can cause behaviours of concern and distress among these individuals. In several health systems, oral healthcare services prioritising these persons are commonly provided under the domain of paediatric dentistry, hospital dentistry, and special care dentistry (4). Each person presents with their unique set of experiences and challenges. Adults with disabilities thus experience varying extents of impact on their ability to receive oral healthcare and/or maintain their oral health. However, specialty care is limited by availability, and not all individuals will require specialist care, such as hospital-based facilities, advanced training in managing complex medical or behavioural needs, and access to general anaesthesia. To stratify oral healthcare services more appropriately to everyone's needs, care is often available at different levels of the healthcare system, mainly within primary/community care, and tertiary/hospital care. Patients experiencing disabilities that profoundly impact on their ability to receive or maintain their oral health are usually referred on to specialist-level services, typically situated within tertiary/hospital care (5).

Adults with disabilities referred for specialist-level oral healthcare services present with significant barriers to receive care, often due to challenges with cooperation or communication. When needed, the oral healthcare practitioner may prescribe the use of advanced pharmacological behavioural management strategies, such as sedation, and general anaesthesia. To understand the complexity of care of patients seen within specialist-level services, several studies have analysed hospital records to describe oral health status, treatment needs, and the frequency of prescribing advanced pharmacological behavioural management (5–8). Moreover, these audits allow for evaluation of services and improvement of resource allocation (9). In these studies, case mix tools are employed to provide an aggregated descriptive of the complexity profile of the patient in addition to their oral health needs. One such example is the British Dental Association (BDA) case mix tool (10) that captures domains of communication, cooperation, medical status, oral risk, access to care, and legal and ethical barriers to care classified according to different levels of complexity. Another case mix tool is the Universal Case Mix Tool (11) that focuses on the accommodations (e.g., need for oral health management under general anaesthesia due to highly challenging behavioural profiles) required for the patient to safely receive care.

In Singapore, specialist-level oral healthcare for adults with disabilities is available within Special Care Dentistry (SCD) services at two national specialty centres for dentistry. These services receive referrals from community dental clinics and other healthcare partners, with available government subsidies to reduce out-of-pocket costs including for those attending specialty services. At present, only a limited number of studies such as those cited above have analysed hospital records of patients with disabilities requiring specialist-level oral healthcare using a case mix tool, with none yet conducted in Singapore. Additionally, the integration between specialist-level oral healthcare services and potential referral sources is not clearly understood in the literature, of which such findings could potentially reveal a systemic barrier in access to care. With a highly integrated public healthcare system and structured referral pathways, this system provides a distinct environment to study patient flow from primary to tertiary care. Therefore, the primary aim of this retrospective audit of hospital records is to examine the profile of adults with disabilities referred for specialist-level care at an SCD service in Singapore, including oral health needs and their referral sources. The secondary aim is to explore associations between patient characteristics and service-related outcomes, including the need for advanced pharmacological behavioural management, to inform service planning and resource allocation.

## Methodology

This study was conducted using hospital records from the National University Centre for Oral Health Singapore (NUCOHS). NUCOHS is one of two national specialty centres for oral health in the country, and is part of the public oral healthcare system, integrated with polyclinic dental services (i.e., primary care dental clinics) and hospital dental clinics. Individuals seeking subsidised dental care can do so through a referral system, often through the polyclinic dental services (12). The centre is also housed within the National University Health System, which is an integrated academic health system and one of Singapore's three public healthcare clusters.

First visit hospital records of patients with appointments tagged as “First Visit Special Needs Dentistry” (note: patients ≥65 years old would be referred on for “First Visit Geriatric Dentistry”, which is not the primary group of interest in this audit) from March 2022 to December 2024 were acquired through the electronic health record (Epic Systems, Wisconsin, United States) of the National University Centre for Oral Health Singapore. Records included structured fields such as referral source, medical diagnoses in International Classification of Diseases (ICD) codes at the time of first visit, aggregated oral status from odontogram charting (presence of caries; presence of at least one tooth with mobility grade II or more), and clinician written notes of the first visit. The primary outcome was whether the indication for advanced pharmacological behavioural management (i.e., sedation, general anaesthesia) was advised or ordered. This was operationalised as a composite binary variable, as the requirement of either of these modalities would necessitate that the patient be managed under a specialist within the SCD service, rather than a generalist or dental officer, which would influence resource allocation. Records retrieved were anonymised and de-identified by an independent third-party before handing over to the study team.

Records of adults with disabilities were included in the sample if they met the inclusion criteria:

- Any patient aged <65 years referred for “Special Needs Dentistry” services
- Presents with disabilities as classified by the SG Enable classification (13) i.e., intellectual, autism, physical disability, or syndromes (e.g., Down Syndrome). Mixed disability was defined as the presence of two or more co-existing disability types documented in the clinical record.
- Referred for specialist dental services

All first visit records meeting the inclusion criteria within the study period were consecutively included to minimise selection bias within the constraints of a retrospective audit. Records were excluded from the sample if the patient presented with medical conditions but without a disability as defined above. Erroneous referrals were also excluded (e.g., an abled young adult seeking dental treatment).

Two reviewers (GKYL, CMT) familiar with the use of the BDA case mix tool scored each case independently. The case mix tool was selected for its comprehensive domains covering a dental patient's ability to communicate and cooperate, and their medical status, oral risk, access to care, and legal and ethical considerations that aligned with the study's aim to create a holistic patient profile. The domain of Medical Status was scored from ICD codes, while Oral Risk Factors was scored from the oral health data and written notes. Domains of Communication, Cooperation, Access to Care, and Legal and Ethical Barriers were scored from the written notes. Consistent with the methodology published by Alkindi in 2016 (6), if complexity related to any domain was not identifiable, it was assumed to not be a concern or issue for the patient and hence scored as “0″. However, this approach may underestimate complexity in cases where documentation was incomplete. Differences in scoring in a domain were also discussed between reviewers until a consensus score was agreed upon. Due to the retrospective nature of the audit and reliance on routine clinical documentation, formal inter-rater reliability was not assessed and is acknowledged as a limitation. The total score would then classify the record into the corresponding incremental complexity levels: *Standard Patient, Some Complexity, Moderate Complexity, Severe Complexity, Extreme Complexity*.

Descriptive statistics (median and interquartile range for quantitative variables; frequency and percentage for qualitative variables) were used to summarise information collected. To evaluate the association of referral source with patients' characteristics and their oral health status, Kruskal–Wallis test for quantitative variables and exact test for qualitative variables were carried out. To examine factors associated with advanced pharmacological behaviour management, logistic regression analysis was performed. Analyses were conducted using available data without imputation. Missing or undocumented variables were treated as absent for descriptive analyses. Statistical significance level was set at *p* < 0.05. All statistical analysis were performed using R 4.4.3 (R Core Team, 2025).

Ethical approval through exemption was granted by the National Healthcare Group Domain Specific Review Board (NHG-DSRB-2024-4362) as this study was conducted as a retrospective audit of de-identified hospital records.

## Results

276 first visit records were retrieved across a 33-month window, of which 184 met the inclusion criteria; 92 records were excluded due to erroneous referrals or if the patient did not present with a qualifying disability diagnosis as detailed in the inclusion criteria.

37.5% were female, and the median age was 26 years (IQR 21–36) (Table 1). Those aged 19–34 years old represented the largest proportion with 60.3%. Referral sources were mainly from tertiary medical services (43.9%) and primary care (40.5%), and 10% were referred from paediatric services. Mixed disability was the most common disability (34.8%), before intellectual disability (28.8%) and autism spectrum disorder (17.9%).

**Table 1:** Demographic of patients referred for special needs dentistry (*N* = 184).

Patient Characteristics	***n* (%)**
Gender	
Male	115 (62.5%)
Female	69 (37.5%)
Age	
15 to 18 years old	24 (13.0%)
19 to 34 years old	111 (60.3%)
35 to 49 years old	30 (16.3%)
50 to 64 years old	19 (10.3%)
(Q1, Q3)	26 (21, 36)
Ethnicity	
Chinese	140 (76.1%)
Malay	21 (11.4%)
Indian	13 (7.1%)
Others	10 (5.4%)
Referral Source	
Primary care	73 (40.5%)
Tertiary centres	79 (43.9%)
Paediatric services	18 (10.0%)
Walk-in	10 (5.6%)
Unknown	4
Disability type	
Mixed disability	64 (34.8%)
Intellectual disability	53 (28.8%)
Autism spectrum disorder	33 (17.9%)
Developmental syndromes	21 (11.4%)
Physical disability	13 (7.1%)

33.2% of patients presented with at least 1 tooth with caries, with a mean DMFT (Decayed, Missing, and Filled Teeth) of 6 (SD 7) (Table 2). 58% did not receive any procedures on the first visit, while around one quarter received scaling, 10% for a restoration, and 4.9% for extraction. Advanced pharmacological behavioural management was indicated for 68 cases (37%).

**Table 2:** Oral health status and needs of patients referred for special needs dentistry.

Oral health characteristics	**n (%)**
DMFT	
Mean (SD)	6 (7)
Median (Q1, Q3)	4 (0, 10)
At least one tooth with caries	
No teeth with caries	123 (66.8%)
At least 1 tooth with caries	61 (33.2%)
At least one tooth with moderate/severe mobility	
Teeth with mobility 1 or less	175 (95.1%)
At least 1 tooth with mobility 2 or more	9 (4.9%)
At least one tooth filled	
No teeth with filling	141 (76.6%)
At least 1 tooth with filling	43 (23.4%)
Type of procedure done	
No procedures	106 (57.6%)
Application only	6 (3.3%)
Scaling	44 (23.9%)
Restoration	19 (10.3%)
Extraction	9 (4.9%)
Advanced pharmacological behavioural management needed	
No	116 (63.0%)
Yes	68 (37.0%)

In terms of case complexity, 89 cases were classified as “Severe Complexity” (48.4%), and 20 cases were of “Extreme Complexity” (10.9%) (Table 3).

**Table 3:** BDA case mix composition (*N* = 184).

BDA case mix criteria	***n* (%)**
Communication	
0	12 (6.5%)
A	30 (16.3%)
B	140 (76.1%)
C	2 (1.1%)
Cooperation	
0	44 (23.9%)
A	62 (33.7%)
B	63 (34.2%)
C	15 (8.2%)
Medical Status	
0	138 (75.0%)
A	1 (0.5%)
B	45 (24.5%)
C	0 (0%)
Oral Risk	
0	3 (1.6%)
A	22 (12%)
B	115 (63%)
C	44 (24%)
Access	
0	8 (4.3%)
A	175 (95.1%)
B	1 (0.5%)
C	0 (0%)
Legal & Ethics	
0	17 (9.2%)
A	0 (0%)
B	167 (90.8%)
C	0 (0%)
Total BDA case mix Score	
Mean (SD)	22 (7)
Median (Q1, Q3)	22 (17, 27)
BDA case mix Complexity Classification	
Standard patient	2 (1.1%)
Some complexity	8 (4.3%)
Moderate complexity	65 (35.3%)
Severe complexity	89 (48.4%)
Extreme complexity	20 (10.9%)

In bivariate analysis (Table 4), the referral source was significantly associated with indication for advanced pharmacological behaviour management (*p* = 0.006). Patients referred from paediatric services had the highest odds compared to primary care referrals (OR = 2.71, 95% CI = 0.94–8.53). Cases with intellectual disability and autism were also more likely to require advanced pharmacological behaviour management (OR = 1.25, CI = 0.52–3.01), although not statistically significant. In the multivariable logistic regression analysis (Table 5) these associations were not statistically significant after adjustment for covariates, although the direction of effect remained similar for certain groups—those with Intellectual Disability were 2.07 (95% CI = 0.63–7.19) more likely, although non-significant, to require advanced pharmacological behavioural management compared to those with autism, controlling for gender, age, ethnicity, oral health status, and BDA complexity.

**Table 4:** Bivariate analysis of patient characteristics associated with requiring advanced pharmacological behavioural management.

Patient characteristics	Not requiring (*N* = 116)	Requiring (*N* = 68)	**Unadj. OR**	**95% CI**	***p*-value**
	**n (%)**	**n (%)**			
Gender					0.635
Male	71 (62%)	44 (38%)	1.00	—	
Female	45 (65%)	24 (35%)	0.86	0.46, 1.60	
**Age (Median, IQR)**	27 (21, 38)	25 (23, 33)	0.99	0.96, 1.01	0.411
**Ethnicity**					0.503
Chinese	85 (61%)	55 (39%)	1.00	—	
Malay	15 (71%)	6 (29%)	0.62	0.21, 1.62	
Indian	8 (62%)	5 (38%)	0.97	0.28, 3.05	
Others	8 (80%)	2 (20%)	0.39	0.06, 1.61	
**Referral Source**					**0**.**006**
Primary care	42 (58%)	31 (42%)	1.00	—	
Walk-in	6 (60%)	4 (40%)	0.90	0.22, 3.43	
Tertiary centres	59 (75%)	20 (25%)	0.46	0.23, 0.91	
Paediatric services	6 (33%)	12 (67%)	2.71	0.94, 8.53	
Unknown	3	1			
**DMFT (Median, IQR)**	4 (0, 10)	3 (0, 9)	0.97	0.93, 1.01	0.166
**At least one tooth with caries**					0.264
No teeth with caries	81 (66%)	42 (34%)	1.00	—	
At least 1 tooth with caries	35 (57%)	26 (43%)	1.43	0.76, 2.69	
**At least one tooth with moderate/severe mobility**					0.330
Teeth with mobility 1 or less	109 (62%)	66 (38%)	1.00	—	
At least 1 tooth with mobility 2 or more	7 (78%)	2 (22%)	0.47	0.07, 2.02	
**At least one tooth filled**					0.072
No teeth with filling	84 (60%)	57 (40%)	1.00	—	
At least 1 tooth with filling	32 (74%)	11 (26%)	0.51	0.23, 1.06	
**Disability type**					**0**.**002**
Autism spectrum disorder	18 (55%)	15 (45%)	1.00	—	
Intellectual disability	26 (49%)	27 (51%)	1.25	0.52, 3.01	
Physical disability	11 (85%)	2 (15%)	0.22	0.03, 0.98	
Developmental syndromes	19 (90%)	2 (10%)	0.13	0.02, 0.53	
Mixed disability	42 (66%)	22 (34%)	0.63	0.27, 1.49	
**BDA case mix Complexity Classification**					**0**.**009**
Standard patient	2 (100%)	0 (0%)	0.00		
Some complexity	7 (88%)	1 (13%)	0.21	0.01, 1.55	
Moderate complexity	49 (75%)	16 (25%)	0.49	0.17, 1.44	
Severe complexity	46 (52%)	43 (48%)	1.40	0.53, 3.89	
Extreme complexity	12 (60%)	8 (40%)	1.00	—	

Bold values indicates statistical significance (*p* < 0.05).

**Table 5:** Multivariable logistic regression for odds of requiring advanced pharmacological behavioural management.

Variable	**Adj. OR**	**95% CI**	***p*-value**
**Gender**			0.427
Male	1.00	—	
Female	0.74	0.34, 1.56	
**Age**	1.03	0.98, 1.07	0.227
**Ethnicity**			0.353
Chinese	1.00	—	
Malay	0.72	0.21, 2.22	
Indian	1.20	0.29, 4.64	
Others	0.21	0.02, 1.27	
**Referral Source**			0.159
Primary care	1.00	—	
Walk-in	0.86	0.19, 3.70	
Tertiary centres	0.73	0.29, 1.79	
Paediatric services	3.31	0.89, 14.0	
Unknown			
**DMFT**	0.93	0.85, 1.01	0.080
**At least one tooth with caries**			0.059
No teeth with caries	1.00	—	
At least 1 tooth with caries	2.34	0.97, 5.89	
**At least one tooth with moderate/severe mobility**			0.446
Teeth with mobility 1 or less	1.00	—	
At least 1 tooth with mobility 2 or more	0.48	0.05, 3.02	
**At least one tooth filled**			0.970
No teeth with filling	1.00	—	
At least 1 tooth with filling	1.02	0.35, 2.91	
**Disability type**			**0**.**017**
Autism spectrum disorder	1.00	—	
Intellectual disability	2.07	0.63, 7.19	
Physical disability	0.43	0.05, 2.81	
Developmental syndromes	0.19	0.02, 1.03	
Mixed disability	0.64	0.20, 2.08	
**BDA case mix Complexity Classification**			0.094
Standard patient	0.00		
Some complexity	0.29	0.01, 3.19	
Moderate complexity	0.39	0.11, 1.37	
Severe complexity	1.19	0.40, 3.70	
Extreme complexity	1.00	—	

Bold values indicates statistical significance (*p* < 0.05).

## Discussion

The oral health status of adults with disabilities within this study appears consistent with community-based findings in Singapore, with Teo et al. reporting a DMFT 6.6 from a single-site sample of a disability centre (14). When compared with tertiary services internationally, such as in Dublin, Ireland (6), and Perth, Australia (7), the proportion of *Severe Complexity* and *Extreme Complexity* profiles in this sample were higher and more skewed towards greater levels of complexity, which may reflect referral patterns and case selection within a tertiary care setting. Similar observations have been reported in these abovementioned settings, where specialist services largely manage patients with greater medical, behavioural, and access-related challenges, consistent with referral-based models of care.

### High oral health needs and case mix complexity

As outlined above, the higher proportion of complex cases in Singapore, when compared to other countries, may reflect referral patterns within the healthcare system where patients with higher levels of complexity are more likely to be referred to specialist services. In special care dentistry practice, first visits usually focus on history taking, assessments, and acclimatisation (15) rather than procedural treatment, potentially explaining why more than half the sample did not undergo any procedures in the first visit. 37% were indicated for advanced pharmacological behavioural management, similar to findings within hospital settings by Schnabl et al. (8) (33%) but lower than Alkindi et al. (6) (55.7%). While the reason for these differences is not explored in this study, the frequency of referrals for management, such as general anaesthesia, remains of importance to hospital services as it informs resource allocation, cost considerations, and workforce capabilities. Without these modalities readily available, adults with disabilities may experience deterioration of untreated oral diseases, or risk acute flare-ups, which can have severe implications on their general health and wellbeing.

Integration and establishing clear care pathways with referral sources are a core function of specialist-level services. Systemic factors, such as the lack of referral pathways for adults with disabilities, have been cited as a major barrier in accessing the appropriate levels of care (16). In this study, referral source and disability type were significantly associated with the need for advanced pharmacological behavioural management in the bivariate analysis. In particular, patients referred from paediatric services demonstrated a higher likelihood of requiring advanced pharmacological behavioural management. Ensuring transition of care for children “ageing out” of paediatric services and into the care of the appropriate level of SCD services has been highlighted in recent years (17, 18). This has been underscored by evidence demonstrating that patients lost to follow-up experience rapid oral heath deterioration in adulthood despite many years of paediatric oral healthcare (19). Our results also suggest that individuals with intellectual disability may also be more likely to require these advanced modalities more than other disability types. This may be due to poorer communication levels and behavioural challenges often compounded by past experiences. In Singapore, disability services usually cater to a cluster of disability-types, such as MINDS, an organisation that provides day care and adult disability home services for adults with higher levels of intellectual disability (20). Hence this study illuminates potential areas of focus for specialty/tertiary oral health services to consider closer integration across the system within their strategic initiatives, namely, 1) vertical integration with community-based intellectual disability organisations (e.g., MINDS) and 2) horizontal integration with tertiary paediatric health and dental services. By facilitating this, patients with specific needs may be better identified and referred onward for care before experiencing further oral health deterioration.

The value of analysing health records (21) is demonstrated in this study of adults with disabilities in a specialty dental centre, describing treatment needs, procedural output, and sub-populations that are more likely to require greater amounts of resources. This allows for hospitals and health systems to consider resource allocation and skill-mix development. This promotes evidence-based initiatives and programmes to improve care pathways and informs workforce development such as in the training of oral healthcare professionals in certain disabilities or person groups. While this study was conducted in a single centre, it also highlights the social, cultural, and systemic identities that each hospital serves—strategies to facilitate care for adults with disabilities should therefore always consider a fit-for-purpose model by examining their own contextual nuances.

### Limitations

Due to the study being an audit and in a single centre, the sampling is limited to available data. As such, the findings are subject to referral and selection bias, whereby individuals with greater complexity, behavioural challenges, or unmet care needs are more likely to be referred. The study also did not include other profiles of patients seen by SCD services such as patients with geriatric conditions and adults without disabilities requiring hospital dentistry, which can influence the proportion of adults with disabilities and the prioritisation of strategic initiatives discussed earlier. The authors further acknowledge a potential bias introduced in the reviewer scoring of the case mix complexity of patient records—this was limited by expertise within the centre in special care dentistry and familiarity with the BDA case mix tool. Formal inter-rater reliability was not quantified, and consensus-based resolution of discrepancies may introduce subjectivity. These factors reflect the constraints of retrospective record-based audits and highlight the importance of cautious interpretation. Nonetheless, the 33-month window of data collected in this study provides a valuable overview of the types of patients seen in the specialist-level service and highlights sub-populations that likely require greater integration of services to increase utilisation.

### Future directions

Following on from this retrospective study, a multi-centre study including Singapore's other national specialty centre and hospital dental clinics with Special Care Dentistry services will create a more comprehensive picture of the healthcare system. Population-based surveys can also provide a more representative picture of the care needs of this population. Furthermore, progressing to a prospective study avoids limitations of retrospective records, and planning could include incorporation of qualitative data including interviews with patients, families, and community-based healthcare providers to understand the barriers to care from their perspective.

## Conclusion

Analysis of hospital records and the use of case mix tools are valuable in evaluating special care dentistry (SCD) services. Within this tertiary referral population, 37.0% required advanced pharmacological behavioural management (e.g., general anaesthesia). BDA case mix complexity levels of Severe (48.4%) and Extreme (10.9%) account for a large proportion of patients referred on to specialist-level SCD services. Nonetheless, the adult disability population is not homogenous and varies in oral health needs and barriers to accessing care across sub-populations. Population segment-specific strategies should be considered when planning special care dentistry interventions, such as for children “ageing out” of paediatric services and adults with intellectual disability. Ultimately, this audit provides the foundational evidence needed to advocate for targeted resource allocation and policy development, providing supporting evidence to inform efforts aimed at improving integration of care pathways for these population segments before their oral health deteriorates.

## Data Availability

The data analyzed in this study is subject to the following licenses/restrictions: The dataset is of patient hospital records and cannot be made available due to confidentiality and personal data privacy restrictions. Requests to access these datasets should be directed to the corresponding author.
